# Synthesis of polymeric nanoparticles by double emulsion and pH-driven: encapsulation of antibiotics and natural products for combating *Escherichia coli* infections

**DOI:** 10.1007/s00253-024-13114-5

**Published:** 2024-05-31

**Authors:** Luís André de Almeida Campos, Jaqueline Barbosa de Souza, Hanne Lazla Rafael de Queiroz Macêdo, Joyce Cordeiro Borges, David Nattan de Oliveira, Isabella Macário Ferro Cavalcanti

**Affiliations:** 1 Keizo Asami Institute (iLIKA), Federal University of Pernambuco (UFPE), Av. Prof. Moraes Rego, Cidade Universitária, Recife, PE 123550670-901 Brazil; 2 Laboratory of Microbiology and Immunology, Academic Center of Vitória (CAV), Federal University of Pernambuco (UFPE), Vitória de Santo Antão, PE Brazil

**Keywords:** Antimicrobial efficacy, *Escherichia coli*, Nanostructures, Polymer matrices, Therapeutic delivery

## Abstract

**Abstract:**

The design, development, and obtaining of nanostructured materials, such as polymeric nanoparticles, have garnered interest due to loading therapeutic agents and its broad applicability. Polymeric nanoparticle synthesis employs advanced techniques such as the double emulsion approach and the pH-driven method, allowing the efficient incorporation of active compounds into these matrices. These loading methods ensure compound stability within the polymeric structure and enable control of the release of therapeutic agents. The ability of loaded polymeric nanoparticles to transport and release therapeutic agents on target manner represents a significant advancement in the quest for effective therapeutic solutions. Amid escalating concerns regarding antimicrobial resistance, interventions using polymeric nanostructures stand out for the possibility of carrying antimicrobial agents and enhancing antibacterial action against antibiotic-resistant bacteria, making a new therapeutic approach or complement to conventional treatments. In this sense, the capability of these polymeric nanoparticles to act against *Escherichia coli* underscores their relevance in controlling bacterial infections. This mini-review provides a comprehensive synthesis of promising techniques for loading therapeutic agents into polymeric nanoparticles highlighting methodologies and their implications, addressing prospects of combating bacterial infections caused by* E. coli.*

**Key points:**

*• The double emulsion method provides control over size and release of bioactives.*

*• The pH-driven method improves the solubility, stability, and release of active.*

*• The methods increase the antibacterial action of those encapsulated in PNPs.*

**Graphical abstract:**

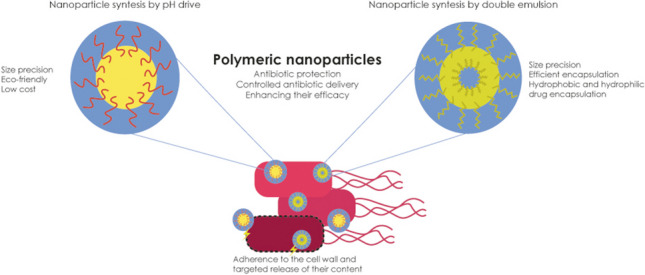

## Introduction

Drug carrier systems present advantages such as reduced toxicity of drug, increased therapeutic efficacy, improved physical and chemical stability of bioactives, and promoted the controlled release of drugs; among the drug transport systems, the main ones are micelles, liposomes, solid lipid nanoparticles, and polymeric nanoparticles (PNPs) (Zielińska et al. 2020; Bhardwaj and Jangde 2023). The encapsulation of natural products and other molecules with biological properties, including conventional antibiotics in PNP, is promising and advantageous strategy. The PNPs are nanocarriers applied orally, nasally, intravenously, intradermally, and intramuscularly, and can be obtained by various techniques that include polymeric precipitation, formation of emulsions, and pH change (Dai et al. 2019; Nwabuife et al. 2022).

Furthermore, in obtaining PNPs, a diversity of synthetic, protein, and saccharide polymers can be used and encapsulate proteins, RNA, DNA, oils, extracts, and a diversity of isolated molecules, protecting therapeutic agents from environmental and physiological degradation (Kreuter 2007; Dai et al. 2019; Nwabuife et al. 2022). All these advantages that are found for the use of PNPs are fundamental for antibacterial therapy, as PNPs can facilitate controlled release at the infection site, resulting in increased drug concentration while reducing side effects. Additionally, it enhances the solubility of poorly soluble compounds in water, allowing for the co-administration of multiple drugs that can interact with various targets, thereby leading to greater therapeutic success and minimizing toxic effects (Osorio-Alvarado et al. 2022; Pan et al. 2022).

In the synthesis of PNPs, controlling the 4S parameters (surface, size, shape, and stiffness) is crucial as they profoundly influence the behavior of nanoparticles in the body and directly impact encapsulation efficiency. In this context, the surface characteristics of these PNPs will determine their interaction with biological components such as proteins and cells, influencing biodistribution and cellular uptake, while the particle size will affect circulation time, tissue penetration, and elimination rates (Castro et al. 2022; Dop et al. 2023). Additionally, the shape of these particles may affect their interactions with biological barriers and cellular uptake mechanisms, potentially impacting therapeutic efficacy. Lastly, stiffness can influence their ability to traverse physiological barriers and efficiently release encapsulated payloads. These methods enhance solubility, stability, and improve therapeutic response (Castro et al. 2022; Molina et al. 2022; Dop et al. 2023).

To obtain PNPs, promising techniques, such as double emulsion and pH-driven, provide physicochemical characteristics, including size, distribution, and drug release profile suitable for applications in disease therapy. These methods offer enhanced precision in tailoring particle attributes compared to older techniques. While older PNP synthesis techniques, such as displacement of solvent followed by evaporation or nanoprecipitation, may present challenges in achieving precise control over particle size and ensuring high encapsulation efficiency (EE%), newer approaches offer more sophisticated to improve the design and obtain the desired characteristics for microbiological applications. However, these older methods still hold relevance when considering their compatibility with specific drugs or formulation requirements (Gaumet et al. 2008; Iqbal et al. 2015; Liu et al. 2020; Ren et al. 2024).

In the face of bacterial resistance, the role of nanobiotechnology emerges as indispensable in the antibacterial therapy. By harnessing the unique properties of PNPs, such as their small size, high surface area-to-volume ratio, and tunable surface chemistry, nanobiotechnology offers innovative strategies to combat resistance mechanisms. Specifically, these PNPs can facilitate targeted delivery of antibacterial agents to bacterial cells, bypassing efflux pumps and other resistance mechanisms that render traditional therapies ineffective (Crosby et al. 2019; Zhang et al. 2022). Additionally, nanobiotechnology enables the development of multifunctional nanomaterials capable of synergistically attacking bacterial pathogens through multiple mechanisms of action, thus overcoming resistance and enhancing therapeutic outcomes. Therefore, the integration of nanobiotechnology into antibacterial therapy represents a pivotal step forward in addressing the pressing challenge of bacterial resistance (Crosby et al. 2019; Zhang et al. 2022).

In this context, this mini-review aims to explore recent advancements and innovations related to the encapsulation of antibiotics and natural products in polymeric nanoparticles (PNPs) to combat *E. coli* infections (Zhou et al. 2023; Zheng et al. 2022) considering articles published between 2019 and 2024. While previous studies have primarily investigated nanoparticles (NPs) as carriers for drug delivery, this review stands out by focusing specifically on promising methodologies to incorporate antibacterial agents into PNPs. Furthermore, it is emphasized that encapsulation in PNPs as a therapeutic approach can overcome the limitations of conventional antibacterial therapies, potentially influencing the epidemiology of *E. coli* infections on a global scale.

## Encapsulation of drugs in nanoparticles

The synthesis of PNPs through conventional methods involves processes such as solvent evaporation and nanoprecipitation. These methods are categorized as bottom-up approaches, starting with the formation of smaller polymeric units, which are subsequently organized and combined, promoting the formation of PNPs with the unique and particle characteristics necessary for the various applications. These conventional methods make it possible to control physicochemical properties including particle size, surface charge, composition, and functionality from the initial stages of synthesis based on the polymer-drug ratio, polymer mass concentration, solvent–water ratio, and water volume (Khorasani et al. 2021; Miranda Calderon et al. 2023).

Considered one of the oldest and most widely used methods, solvent displacement and evaporation involve the formation of PNPs through the displacement and controlled evaporation of a solvent containing polymers. Initially, the polymers are solubilized in an organic solvent (oil phase) to form a homogeneous solution, which is dripped into a liquid medium (aqueous phase). With temperature and pressure control, solvent displacement and gradual evaporation occur, resulting in polymer precipitation and the formation of PNPs (Håkansson and Rayner 2018; Pulingam et al. 2022).

During the evaporation process, the polymer molecules organize themselves to generate nanoscale structures. This conventional method has become relevant due to its characteristics, such as low cost, high EE%, lower complexity, and practicality (Pulingam et al. 2022; Combes et al. 2023). However, it is necessary to develop and understand new methodologies with the same effectiveness, lower cost, practicality, easy reproducibility, scalable, and environmentally sustainable (Combes et al. 2023). Therefore, the selection of the most appropriate methodology will require a thorough analysis considering not only the requirements of the final product but also the applicability, bioavailability, routes of administration, specific targets, and the nature of the drugs and their hydrophilicity and hydrophobicity (Panigrahi et al. 2021).

Although the conventional method presents promising prospects, its adoption is limited due to the high costs of certain equipment and reduced sustainability, resulting in negative environmental impacts and significant energy consumption. Furthermore, from a nanobiotechnological point of view, there are challenges related to particle size uniformity, surface charge, leading to the need for additional steps for the stabilization of PNPs (Zhang et al. 2017; Ma et al. 2023).

## Promising techniques for drug encapsulation

### Double emulsion

Various encapsulation methods, including single emulsion, nanoprecipitation, supercritical fluids, and spray drying, are employed for encapsulating antibiotics in PNPs (Zielińska et al. 2020; Song et al. 2021; Fahmy et al. 2022; Yassin et al. 2023). However, these techniques, especially the single emulsion method, face stability problems in PNPs, such as particle coalescence, rupture of internal droplets, and phase separation. Therefore, the double emulsion (DE) method stands out as promising for encapsulating antibiotics with low water solubility in PNPs (Jamshidifar et al. 2022; Sun et al. 2023b).

**Fig. 1:**
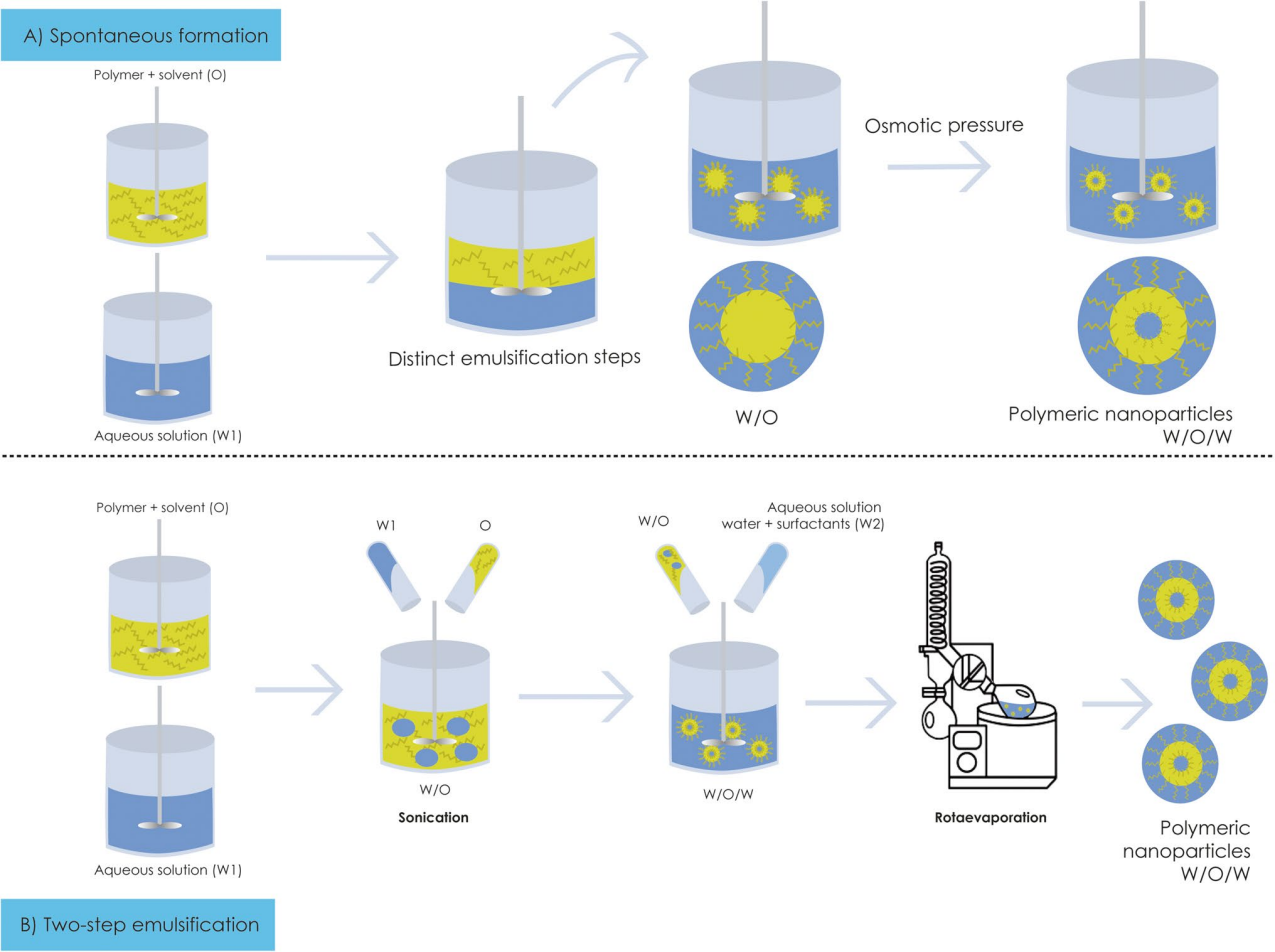
Preparation of polymeric nanoparticles via double emulsion technique. O, oil phase; W1, water phase 1; W2, water phase 2; W/O, oil-in-water; W/O/W, water-in-oil-in-water. Source: created by the authors using Adobe illustrator

PNPs synthesized through DE, also known as water-in-oil-in-water emulsions (W/O/W), consist of complex nanostructure ideas for encapsulating active principles (Fig. 1). In this method, water droplets are dispersed in oil or in oil/polymer globules, which in turn are dispersed in an aqueous phase. This structure has been remarkable in its ability to encapsulate a wide range of molecules, even those that are distinct or incompatible with each other such hydrophobic and hydrophilic molecules, within a single nanosystem (Iqbal et al. 2015; Panigrahi et al. 2021; Song et al. 2021; Fahmy et al. 2022). Synthetic polymers like poly(lactide-co-glycolide) (PLGA) and polyethylene glycol (PEG) (Fahmy et al. 2022), and polycaprolactone (PCL) (Song et al. 2021) are used in the preparation of PNPs.

Previously described by William Seifriz in 1924, DE was the subject of studies to understand how the density of the used oil influenced their formation. It was observed that when straw-oil was used, a coarse oil-in-water emulsion was formed, with oil globules encapsulating a fine water-in-oil emulsion. However, these double droplets quickly became unstable due to osmotic pressure. Nevertheless, despite this, their fragility proved advantageous for controlled release of materials through osmotic pressure, with the first documented application in the 1960s involving insulin encapsulation. Subsequently, techniques were developed using surfactants that enabled an increase in the stability of PNP formulations (Seifriz 1925; Engel et al. 1968; Heidari et al. 2022).

Two main methods are employed for the synthesis of PNPs by DE; the first is spontaneous formation, which occurs naturally, without the need for distinct emulsification steps. In this technique, DEs are generated simultaneously, exploiting phase compatibility to induce PNP formation. Although simpler, this technique may offer less control over particle size characteristics and EE% (Clegg et al. 2016). The second method is two-step emulsification that involves the solubilization of the hydrophilic drug in the aqueous phase, followed by the addition of lipophilic polymers and/or drugs in an organic solvent. These phases are homogenized to form a W/O emulsion, stabilized by surfactants. In a subsequent step, this previously formed emulsion is combined with another aqueous phase and an emulsifier, followed by the removal of the organic solvent. The produced PNPs can be isolated through ultracentrifugation and proper washing. This method provides greater control over particle size, distribution, and properties, allowing for more precise adjustments during each stage of the manufacturing process (Iqbal et al. 2015; Panigrahi et al. 2021).

Simon et al. (2020) conducted a study to obtain vancomycin PNPs using the DE method. In the first step, the emulsion was obtained by mixing an aqueous solution of antibiotic (vancomycin) and a solution of organic solvents dichloromethane and ethyl acetate with the polymer (PLGA). This mixture was kept under magnetic stirring in the presence of an ultrasound probe. The second emulsion was obtained by mixing the first emulsion in aqueous solution containing surfactant polyvinyl acetate (PVA) or didecyldimethylammonium bromide (DMAB), under the same conditions used for the first emulsification. The second emulsion was then added to an aqueous solution of PVA or DMAB to facilitate further dispersion of PNPs in the aqueous phase. PNPs were concentrated, and the organic solvent removed under reduced pressure. PNPs are collected from the final emulsion by centrifugation followed by resuspension in ultrapure or sterile water. These suspensions were used directly or collected by a second centrifugation and resuspension in water. In some cases, vancomycin-loaded PNPs were flash frozen in liquid nitrogen and freeze dried at − 60 °C and 80 μHg.

Similarly, gentamicin was encapsulated in PLGA PNPs by the DE method followed by solvent evaporation by Sun et al. (2021). Initially, the polymer (PLGA) was dissolved in organic solvent (dichloromethane) constituting the oil phase in DE. The gentamicin was dissolved in distilled water and mixed with surfactant (PVA) solution, constituting the water phase 1 (W1). PVA solution was used as the water/aqueous phase 2 (W2). First, the oil phase was mixed with W1 for making a primary emulsion solution, and the mixture was sonicated at 35% amplitude yielding a primary emulsion solution. This was to disperse the mixture to have small nanodroplets. The primary emulsion was mixed with W2, leading to a double emulsion of nanodroplets. After the double emulsion solution was stirred for 4 h, PNPs precipitated after the dichloromethane had diffused from oil phase to W2 resulting in the PLGA PNPs precipitating around W1. The PVA remained at the interface during the diffusion process and helped PNPs encapsulate antibiotics. The PLGA PNPs were formed after the solvent was completely diffused to W2. The emulsions were centrifuged at 6000 rpm for 10 min and rinsed with 5 ml dissolute water to wash away any residue of nonparticulate PLGA or PVA (Sun et al. 2021).

Table 1 compiles the main studies that investigated drug encapsulation in PNPs using the DE technique. The listed articles provide details about the materials used, the encapsulated drugs, and various relevant parameters, such as particle size, polydispersity index, zeta potential, and EE%. These details play a crucial role in understanding the variables and results obtained during the encapsulation process, significantly contributing to advancing knowledge in this specific research field.

**Table 1:** Drug encapsulation studies in PNPs via double emulsions

Materials	Drug	Size, polydispersity index, and zeta potential	EE (%)	Reference
PLGA, dichloromethane, and PVA	Epirubicin	169.0–297.6 nm 0.003–0.055 − 10.9 to − 25.2 mV	PVA: 21.96–38.18%	Esim et al. (2020)
PLGA, dichloromethane, and poloxamer 188		318.9–417 nm 0.181–0.324 − 11.0 to − 32.4 mV	20.99–39.4%	Esim et al. (2020)
PLGA, ethyl acetate, PCL, chloroform, and poloxamer	Meloxicam	142 nm 0.276 − 16.2 mV	71.76%	Akel et al. (2021)
Distilled water, DMSO, PLGA, pluronic F-127, and PVA	Doxorubicin/ Erlotinib	237.7 nm 0.087 − 1.7 mV	Doxorubicin: 92.7% Erlotinib: 96.1%	Lee et al. (2021)
PLGA, Milli-Q water, sodium cholate, pDNA, dichloromethane (DCM)	pDNA	88–93 nm ND − 57.32 ± 10.16 mV	33.16 ± 20.81%	López-Royo et al. (2021)
Chitosan, dichloromethane, span 80, PVA and glutaraldehyde	Capreomycin	343.7–826.9 nm 0.005 − 0.76 to − 1.5 mV	17.3–62.3%	Alenazi et al. (2022)
PLGA, dichloromethane ethyl acetate, and PVA	Vancomycin	210.5 nm 0.08, − 22 mV	46%	Simon et al. (2020)
PLGA, dichloromethane ethyl acetate, and DMAB		83.4 nm 0.16 + 27.0	62%	Simon et al. (2020)

*PLGA*, poly(lactide-co-glycolide); *PVA*, poly (vinyl alcohol); *PCL*, poly(ε-caprolactone); *pDNA*, plasmid DNA;

*DMAB, didecyldimethylammonium bromide; DE, double emulsion; ND, not determined*

Created by the authors (2023)

Some parameters are fundamental for the synthesis of PNPs, including solvents, polymers, and surfactants. In that regard, solvents play distinct roles, from dissolving components to forming emulsions, essential for the successful synthesis of PNPs with desired properties, where chloroform, dichloromethane, ethyl acetate, distilled water, and Milli-Q water are commonly described for these purposes. Chloroform and dichloromethane are frequently used for their ability to dissolve polymers, though their toxicity and volatility demand careful handling, while ethyl acetate, with similar properties, is considered less toxic (Akel et al. 2021; Alkholief et al. 2022; Ding et al. 2019). On the other hand, distilled water is employed as an aqueous phase to dissolve hydrophilic compounds, while Milli-Q water, renowned for its purity, is crucial in synthesis steps, ensuring high-quality particles (Haggag et al. 2018; Lee et al. 2021).

PLGA, PCL, and chitosan are often chosen as polymers in the synthesis of PNPs using the DE technique due to their versatile properties and individual advantages. PLGA is widely favored for its biocompatibility, biodegradability, and ability to release compounds in a controlled manner, On the other hand, PCL provides a slower degradation rate and is known for its stability, making it suitable for sustained release systems. Chitosan, a natural polymer derived from chitin, possesses notable biological properties such as biocompatibility and the ability to penetrate cells. Its use enables the formulation of PNPs with potential for targeted drug delivery to specific tissues, along with its capability to interact with biological molecules, making it an appealing choice for biomedical applications (Iqbal et al. 2015; López-Royo et al. 2021; Akel et al. 2021; Alenazi et al. 2022; Aboelenin et al. 2024).

Since the stability of DE is a key parameter in the quality of these PNPs, many formulation strategies have been developed to increase their stability. Therefore, the choice of surfactants to be used must be made to ensure the stability of each independent emulsion. For water-in-oil (W/O) primary emulsions, it is preferable to use a surfactant with a hydrophilic-lipophilic balance (HLB) value below 7, usually around 3–4, to prevent instability of the internal droplets through coalescence and flocculation (Sousa et al. 2022). Additionally, to stabilize the external surface, a surfactant with an HLB value above 10 is required, as these are preferred to stabilize the interface between the phases, promoting the stability of PNPs. This stabilization is crucial to maintain the integrity of the double emulsion, preventing coalescence or phase separation during subsequent storage or processing (Marhamati et al. 2021; Majid et al. 2024).

Furthermore, the presence of surfactants directly impacts the ability of PNPs to effectively encapsulate the drug, directly influencing the EE% through a) stabilization of interfaces, maintaining NP stability, and ensuring retention of the encapsulated material; b) compatibility of the encapsulated material, because they can improve affinity between phases and the encapsulated active ingredient, resulting in higher EE%; and c) reduction of losses since during processing, they can keep PNPs stable and minimize premature release of the encapsulated material, thus contributing to higher EE% (Yekeen et al. 2020; Cortés et al. 2021).

The non-ionic surfactants, such as polysorbates (Tween 20, Tween 80) and sorbitan esters (Span 20, Span 80), and polyvinyl alcohol are frequently used to ensure stability at the water–oil and oil–water interfaces (Akel et al. 2021; Alenazi et al. 2022; Lee et al. 2021). Additionally, polyethylene glycol derivatives, like Triton X-100, are widely utilized due to their emulsifying properties (López-Royo et al. 2021). Amphiphilic surfactants such as poloxamers also play a fundamental role in providing stability and controlling the size of PNPs during the double emulsion synthesis process (Esim et al. 2020).

Based on this principle of the stability and controlling the size of PNPs, Esim et al. (2020) synthesized PLGA PNPs loaded with epirubicin using emulsifiers such as PVA (HLB 18) and poloxamer 188 (HLB: 29). They noticed variations in particle size with different surfactant concentrations. Higher concentrations of PVA, from 0.3 to 1%, notably reduced particle size (297.6 nm and 230 nm, respectively), but at 2%, an increase was observed (250.7 nm). Increasing surfactant concentration lowered surface tension, leading to smaller particles, however, elevated PVA concentration increased the aqueous phase viscosity, enlarging particles. Poloxamer 188 was less effective than PVA, with sizes of 400.6 nm and 318.9 nm at 0.3% to 1%, respectively. At 2%, size rose to 417 nm due to higher viscosity causing particle aggregation.

The influence of surfactants, such as PVA and poloxamer 188, on PNP EE% is crucial in DE synthesis. Studies have demonstrated that increasing PVA concentration resulted in a significant reduction in particle size and a corresponding increase in drug EE% (Esim et al. 2020; Lee et al. 2021; Alenazi et al. 2022). On the other hand, poloxamer 188 was found to be less effective in reducing particle size, especially at higher concentrations, which contributed to particle aggregation (Esim et al. 2020; Akel et al. 2021). Although both have influenced the EE%, PVA proved to be more efficient, as it presented better results in terms of reducing particle size and increasing EE%, when compared to poloxamer 188. However, the choice of the best surfactant may vary according to the specific objective of the application and the desired properties of the PNPs (Alenazi et al. 2022).

Therefore, the DE method stands out as an invaluable technique in the development of PNPs, especially when aiming to encapsulate antibacterial agents within polymeric nanoparticles. Its unique ability to efficiently encapsulate molecules with antibacterial activity, regardless of their hydrophilic, lipophilic, or amphiphilic nature, underscores its fundamental utility for targeted therapeutic strategies against bacterial infections. The versatility of this approach, combined with its simplicity and cost-effectiveness, not only enables the creation of multifunctional systems but also opens up promising avenues for combating antibiotic-resistant bacteria and other infectious pathogens in healthcare applications (Heidari et al. 2022; Aboelenin et al. 2024; Majid et al. 2024).

### pH-driven

The increase in the complexity of techniques associated with the development of drug delivery systems has become evident, with innovative ways of synthesizing protein-based PNPs emerging. In this sense, the pH-driven method emerges as an alternative to mitigate some disadvantages of conventional techniques, distinguishing itself mainly by not requiring the use of organic solvents and by the need for robust equipment in the NP synthesis process (Dai et al. 2019; Wang et al. 2020; Ye et al. 2022).

The pH-driven method relies on the ability of encapsulated active to solubilize and stabilize within the medium, through adjustments in pH values (Li et al. 2022; Zhang et al. 2023). Additionally, this technique excels in allowing thermal stability, resulting in prolonged integrity of the encapsulated medication under high-temperature conditions. These distinctive features position this method as an innovative alternative, fostering significant advancements in obtaining PNPs (Dai et al. 2019; Zhan et al. 2020; Wei et al. 2021). Thus, the prospects for durability and therapeutic efficacy of encapsulated medicines are expanded, especially in protein-based nanoparticles (Yuan et al. 2022a, b).

**Fig. 2:**
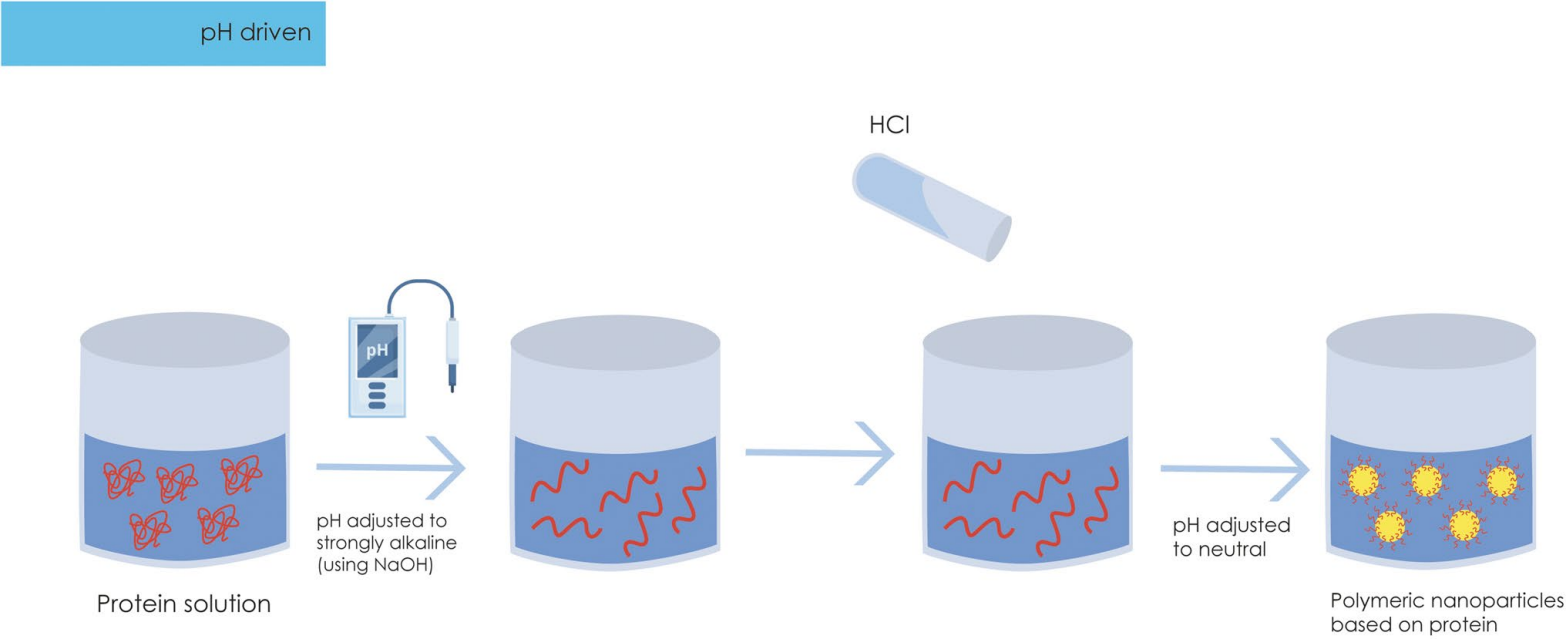
Preparation of polymeric nanoparticles via pH-driven method. NaOH, sodium hydroxide; HCl, hydrochloric acid. Source: created by the authors using Adobe illustrator

For the synthesis of PNPs using this technique, there are two main approaches: I) polymers and proteins are dispersed in the aqueous phase and adjusted to an alkaline state using NaOH; II) polymers are dispersed in the aqueous phase to be adjusted to an alkaline state with NaOH, followed by the pre-treatment of lipids and surfactants for NP formation. In both situations, these undergo magnetic agitation, and subsequently, the pH is adjusted close to neutrality using HCl, and any insoluble particles are removed by centrifugation (Fig. 2) (Wei et al. 2021).

The ability to manipulate pH to destabilize and subsequently refold proteins is pivotal in the synthesis of PNPs. This technique allows for the control of protein structure and interactions, resulting in the controlled formation of stable and functional PNPs. When proteins are exposed to extreme pH levels, they may lose their three-dimensional structure, becoming more flexible and prone to molecular rearrangements. Upon readjusting the pH back to neutral, proteins can regain their functional structures through favorable amino acid interactions. This pH-induced refolding enables proteins to spontaneously organize into new conformations, including the formation of PNPs (Wang et al. 2018). In this way, this method utilizes a base (NaOH) and an acid (HCl), necessitating a preliminary investigation to determine whether the compound in question will undergo degradation amid extreme pH variations. Typically, studies are conducted to assess the compound’s degradation in an alkaline environment (Dai et al. 2019).

The procedure for obtaining protein-based nanoparticles using the pH-driven method stands out for its simplicity and cost-effectiveness. Initially, the polymers (proteins) are solubilized in deionized water under magnetic stirring, carried out in an alkaline environment with a pH set at 12.0 with 4.0 mol/L NaOH. To promote the specific formation of the desired PNPs, it is essential to subsequently reduce the pH to 7.0 by using 1.0 mol/L HCl under continuous stirring. This adjustment is crucial to optimize molecular interactions and the stability of the PNPs during their formulation. This method is economically viable, making it an attractive approach for the controlled and efficient production of PNPs for various scientific and technological applications (Zhan et al. 2020; Wei et al. 2021). The highlighted studies in Table 2 focus on encapsulation through the pH-driven technique, providing a comprehensive analysis of the success in PNPs synthesis. By exploring different proteins such as zein and casein, these research efforts demonstrate the versatility of this approach in obtaining nanostructures with noteworthy properties, including stability and EE%.

**Table 2:** Physical–chemical characteristics of PNPs produced by a pH-based method

Materials	Compounds	Size, polydispersity index, and zeta potential	EE (%)	Reference
Cellulose, deionized water	Curcumin	100 nm 0.3 30 mV	> 90%	Yuan et al. (2022a, b)
HPSS, deionized water, NaOH, HCl	Curcumin	119–141 nm 0.58–0.61 ND	> 96%	Sun et al. (2023a)
Zein, whey protein isolate, deionized water, NaOH, HCl	Curcumin	92.4 ± 1.6 nm < 0.3 ∼ − 25 mV	68.13 ± 2.18%	Zhan et al. (2020)
Zein, deionized water, NaOH, HCl, rhamnolipid	Curcumin rhamnolipid	96.2–81.8 nm < 0.4 ∼ − 30 mV	96.2 to 81.8%	Dai et al. (2019)

*HPSS*, soy sauce production; *NaOH*, sodium hydroxide; *HCl*, hydrochloric acid; *ND*, not determined

Source: created by the authors (2023)

This technique is extensively employed in the context of nanoencapsulation, particularly for incorporating lipophilic phenols, primarily curcumin (CUR). This is due to the numerous intrinsic limitations of this polyphenolic compound that constrain its therapeutic application. Through this methodology, efficient nanoencapsulation of CUR is achievable, facilitating the effective delivery of this compound by enabling its solubilization in an aqueous medium through pH variation. The process induces the ionization of the hydroxyl groups within the molecular structure of CUR in response to the pH shift from neutral to alkaline conditions; consequently, the oxygen atoms of these groups acquire a negative charge, significantly enhancing the solubility of CUR in water (Pan et al. 2014; Chen et al. 2023). In this sense, CUR emerges as an excellent candidate for encapsulation, as research substantiates its resistance to alkaline degradation and the stability of polymeric PNPs containing CUR at high temperatures and over an extended storage period after the encapsulation process (Pan et al. 2014; Zhan et al. 2020).

Protein variants, drawn from diverse sources like mung beans (Mohammadian et al. 2021), whey protein, notably sodium caseinate (Peng et al. 2020; Xu et al. 2021), and proteins derived from soy and wheat (Sun et al. 2023a; Kevij et al. 2020), are integral components in the process of nanoencapsulation employing the described method. Zein, valued for its versatility and cost-effectiveness, is a frequent choice as a polymer in fabricating PNPs utilizing the pH-driven technique. Research supports the efficacy of the pH-driven method in producing zein-based nanoparticles (ZNPs), showing favorable outcomes in terms of optimal particle size distribution, substantial zeta potential, and high EE% (Dai et al. 2019; Zhan et al. 2020).

The studies involving zein have yielded promising results, particularly in terms of stability. In an investigation conducted by Dai et al. (2019), the stability of NPs was meticulously examined through exposure to stressors such as temperature elevation within the range of 37 to 55 °C, as well as the submission of these particles to ionic forces. It was observed that, even in the face of considerable temperature fluctuations spanning from 25 to 37 °C, the NPs demonstrated a remarkable ability to preserve CUR over a 1-month period. This finding demonstrates the efficacy of ZNPs produced through the pH-driven method as potential carriers for the stabilization and controlled delivery of bioactive compounds, such as CUR. In analyses conducted by Zhan et al. (2020), the PNPs demonstrated stability at high temperatures (80 °C), with an additional noteworthy increase in aqueous solubility.

Studies by Sun et al. (2023a) and Yuan et al. (2022a, b) present diverse yet effective strategies for encapsulating curcumin (CUR) within nanoparticle systems, each highlighting unique approaches and outcomes. Sun et al. (2023a) used soy soaking by-products to formulate CUR-loaded PNPs through the pH-driven technique. In this study, the authors confirmed the high encapsulation efficiency, homogeneity, and stability of PNPs. Conversely, Yuan et al. (2022a, b) utilized cellulose nanocrystals (CNC) as a one-dimensional matrix for pH-directed CUR encapsulation with high encapsulation efficiency. This system exhibited stability across a pH range of 3.0 to 8.0 due to robust electrostatic repulsions and maintained stability during storage at 25 °C for 4 weeks, effectively delaying CUR degradation. These findings also indicated enhanced CUR bioavailability in the gastric environment, suggesting the potential efficacy of the systems for oral administration.

Various proteins exhibit potent antibacterial activity and are therefore promising candidates for incorporation into such PNPs. For instance, gliadin, lactoferrin, and defensins are well-known antimicrobial proteins that can disrupt bacterial cell walls or interfere with essential microbial processes. Moreover, peptides derived from larger proteins, such as lactoferricin or defensin analogs, possess remarkable antibacterial efficacy against a broad spectrum of pathogens. By encapsulating these antibacterial proteins or peptides within pH-responsive polymeric nanoparticles, their stability can be enhanced, and controlled release can be achieved at specific sites within the body, optimizing therapeutic outcomes (Chen et al. 2021; Rao et al. 2023). This approach not only offers protection to the proteins from enzymatic degradation but also allows for targeted delivery to infection sites, minimizing systemic side effects.

Furthermore, many of these proteins can enhance the antibacterial action when they are used as polymeric constituents in PNPs, and the pH-driven method brings this possibility by promoting emerges as an efficient approach for the production of PNPs in a simplified, safe, and direct manner with absence of heating or the use of organic solvents, coupled with a low cost, a more sustainable synthesis, and effective delivery of diverse antibacterial proteins, enhancing their therapeutic potential in combating bacterial infections (Dai et al. 2019; Zhan et al. 2020; Wei et al. 2021; Zhao et al. 2024).

## Applications of innovative methods for synthesizing PNPs in antibacterial therapy against *Escherichia coli.*

Bacterial infections represent one of the major challenges to global public health due to the persistence of these infections, treatment inefficacy, and consequently, high rates of morbidity and mortality. It is estimated that about 14% of hospitalizations are attributed to bacterial infections. These infections are marked by bacterial resistance, which is the ability to survive even after exposure to antimicrobial agents, and can be developed through reduced cellular permeability, enzymatic inactivation, efflux pump production, alterations in binding sites, as well as biofilm formation, providing greater stability and protection to bacteria (Yu et al. 2020; Coriolano et al. 2021).

As infections caused by Gram-negative bacteria, such as *E. coli* contribute to this issue, being among the main causes of urinary tract infections, gastrointestinal diseases, and systemic infections. Due to antibiotic resistance in strains of this microorganism, such as beta-lactamase enzymes, like extended-spectrum beta-lactamases (ESBLs), carbapenemases, cephalosporinase AmpC, and bla_NDM-1_ gene (Yair and Gophna 2018; Pereira et al. 2019). Considering this versatility to cause a variety of infections, along with constantly evolving resistance mechanisms, it highlights the emerging need for effective innovative therapeutic approaches, such as encapsulating active agents with antibacterial potential in PNPs, capable of acting against this microorganism (Cui et al. 2020; Perveen et al. 2020). Table 3 represents studies that assessed the efficacy of PNPs against *E. coli* isolates.

**Table 3:** Studies on PNPs in therapy against *E. coli* infections

Methodology	Drug	Polymer	Size (nm), polydispersity index, and zeta potential (mV)	EE%	Minimum inhibitory concentration (MIC)	Zone of inhibition	Reference
Double emulsion	Ciprofloxacin	PLGA-PEG	238–318.4 nm 0.125–0.158 ND	56.1–68.4%	0.0188 μg/mL	ND	Song et al. (2021)
Double emulsion	*Peganum harmala*	PLGA-PEG	207 nm 0.17 − 31.60 mV	81%	0.025 mg/mL	ND	Fahmy et al. (2022)
Double emulsion	Gentamicin	PLGA	130 nm ND ND	ND	ND	7.16 mm	Sun et al. (2023b)
pH-driven	Thymol	Casein	79.4 nm ND − 19.4 mV	85.2%	0.312 mg/mL	ND	Zhou et al. (2023)
pH-driven	Carvacrol	Zein and casein	70–200 nm 0.176–0.358 − 29.4 to − 32.7 mV	77.96–82.19%	10 mg/mL	ND	Zheng et al. (2022)

*PLGA*, poly (lactic-co-glycolic acid); *PEG*, polyethylene glycol; *ND*, Not determined

Source: created by the authors (2023)

The studies conducted by Song et al. (2021), Fahmy et al. (2022), and Sun et al. (2023b) explored various nanoparticle formulations and their activity against *E. coli* using the DE technique. Song et al. (2021) observed that ciprofloxacin-loaded nanoparticles displayed a dose-dependent inhibition of bacterial growth, with a MIC of 0.0188 μg/mL, while empty particles showed no significant effect, with an MIC exceeding 10 μg/mL. Fahmy et al. (2022) investigated nanoparticles containing *Peganum harmala* alkaloid complex (HARF), demonstrating an MIC of 0.025 mg/mL, showcasing higher antibacterial efficacy than the free HARF (0.5 mg/mL). Lastly, Sun et al. (2023b) analyzed nanofibers loaded with gentamicin nanoparticles, resulting in an inhibition zone of 7.16 mm, markedly greater than observed with nanoparticles alone without the drug (0 mm).

Based on the obtained results, it is noted that the gradual release of active ingredients encapsulated within polymeric nanoparticles can exert a continuous effect on bacterial growth over time due to the controlled release property that inhibits such growth. Additionally, improvements in physicochemical properties, such as enhanced hydrophilicity upon incorporation into the nanoparticles, may favor bactericidal action (Simon et al. 2020; Sun et al. 2023b). These particles also possess the ability to adhere to the cell wall, as evidenced by their negative surface charge (Table 3), facilitating the targeted release of their content. The crucial relevance of the double emulsion technique in achieving these promising outcomes is highlighted, allowing for precise and controlled formulation of the nanoparticles, enhancing their efficacy as antimicrobial agents (Song et al. 2021; Fahmy et al. 2022; Sun et al. 2023b).

From the perspective of preparing a nanocarrier in an innovative, simple, economical, safe, and low-energy way, PNPs were prepared by a method based on pH (Yuan et al. 2022a, b). The pH-driven technique is a low-energy, solvent-free self-assembly method used in various carrier encapsulations of natural compounds, such as monoterpenes, with antibacterial activity, like for example a carvacrol (CAR) and thymol (Li et al. 2022; Lia et al. 202; Zheng et al. 2022). The antibacterial effects of CAR encapsulated in zein/sodium caseinate nanoparticles against *E. coli* were demonstrated. When the concentrations of the nanoparticles were 0, 2, 6, and 10 mg/mL, the antibacterial rates reached 54%, 72%, and 85%, respectively (Zheng et al. 2022). Studies with other terpenes, such as thymol encapsulated in caseinate nanoparticles, have shown antimicrobial activity against *E. coli* (MIC of 312 µg/mL) (Zhou et al. 2023).

Both thymol and carvacrol impact the bacterial cell membrane, causing damage to its structure and function by mechanisms that modify membrane permeability, resulting in the loss of ions and vital cellular components (Zheng et al. 2022). This culminates in inhibiting the growth of *E. coli* and eventually leading to cell death. However, due to the low stability of these compounds, encapsulation in casein and zein nanoparticles, using the pH-driven method, could be a viable alternative (Zhou et al. 2023). These proteins play a crucial role in forming stable nanoparticles, enhancing the solubility, stability, and controlled release of these compounds. The interaction between these compounds and proteins allows for a more controlled release, increasing exposure to target microorganisms like *E. coli*, thereby augmenting their inhibitory capacity (Zhang et al. 2023).

Other studies highlight the importance of this innovative method for encapsulating phenolic compounds (PCs) (Yuan et al. 2023). In comparison to conventional encapsulation methods of phenolic compounds (PCs), pH-driven methods are green, simple, and require low-energy consumption. It has a huge potential for industrial applications and can overcome more effectively the aqueous solubility, stability, and bioavailability issues related to PCs by changing pH to induce the encapsulation of PCs (Zhang et al. 2022).

PNPs synthesized using the methods described in Table 3 have emerged as a promising strategy to combat *E. coli* infections. The results highlight significant advantages, such as the controlled delivery of therapeutic agents including conventional antibiotics and encapsulated natural compounds, intended for treating these infections. Administering these PNPs, either orally or topically, ensures the maintenance of effective therapeutic concentrations at the infection site while minimizing undesirable side effects and may contribute to increased patient adherence to treatment (Alenazi et al. 2022; Sun et al. 2023b).

## Conclusion and future perspectives

The synthesis of polymeric nanoparticles through the double emulsion method has proven to be highly advantageous, allowing for the creation of particles with adjustable properties. This method provides control over size and controlled release of encapsulated therapeutic agents, making it highly versatile for biomedical applications. Alongside this, the pH-driven method, by promoting the formation of nanoparticles with adjustable surface charges, offers an effective platform for targeted release of antimicrobial agents and enhancing the solubility and stability of the compounds.

The protection conferred by these methods preserves the stability and enhances the efficacy of antimicrobial agents, ensuring targeted release at the site of infection. Specifically concerning its application against *E. coli*, the implementation of methodologies like the double emulsion technique has revealed its potential to form complex structures capable of incorporating more than one drug/natural product. On the other hand, the pH-controlled method induces conformational changes in proteins, facilitating the development of PNPs tailored to combat this microorganism.

These advances in PNP synthesis methodologies contribute to the development of antimicrobial encapsulation systems. These promising methods expand knowledge of techniques for preparing new PNPs and bring future perspectives for various nanobiotechnological applications, enhancing the encapsulation of bioactive molecules in a simpler, low-cost way, without or with few solvents. In particular, these approaches expand the prospects for applications in the microbiological area, directing antibacterial agents to their site of action. Thus, these PNPs become promising, safe, and efficient approaches for the treatment of *E. coli* infections.

## Data Availability

The authors declare that they are aware of the availability of data in my main manuscript file.
